# Patient and Provider Reported Reasons for Lost to Follow Up in MDRTB Treatment: A Qualitative Study from a Drug Resistant TB Centre in India

**DOI:** 10.1371/journal.pone.0135802

**Published:** 2015-08-24

**Authors:** Rajesh D. Deshmukh, D. J. Dhande, Kuldeep Singh Sachdeva, Achuthan Sreenivas, A. M. V. Kumar, Srinath Satyanarayana, Malik Parmar, Patrick K. Moonan, Terrence Q. Lo

**Affiliations:** 1 World Health Organisation, Country Office for India, New Delhi, India; 2 Department of Pulmonary Medicine, Drug Resistant TB centre, Lata Mangeshkar Hospital, Nagpur, Maharashtra, India; 3 Central TB Division, Ministry of Health and Family Welfare, New Delhi, India; 4 International Union against Tuberculosis and Lung Disease, South East Asia Office, New Delhi, India; 5 Division of TB Elimination, International Research Programs Branch, U.S. Centers for Disease Control and Prevention, Atlanta, Georiga, United States of America; 6 Department of Epidemiology and Biostatistics, McGill University, Montreal, Canada; Public Health Research Institute at RBHS, UNITED STATES

## Abstract

**Introduction:**

Multidrug-resistant Tuberculosis (MDR TB) is emerging public health concern globally. Lost to follow-up (LTFU) is one of the key challenge in MDRTB treatment. In 2013, 18% of MDR TB patients were reported LTFU in India. A qualitative study was conducted to obtain better understanding of both patient and provider related factors for LTFU among MDR TB treatment.

**Methods:**

Qualitative semi-structured personal interviews were conducted with 20 MDRTB patients reported as LTFU and 10 treatment providers in seven districts linked to Nagpur Drug resistant TB Centre (DRTBC) during August 2012–February 2013. Interviews were transcribed and inductive content analysis was performed to derive emergent themes.

**Results:**

We found multiple factors influencing MDR TB treatment adherence. Barriers to treatment adherence included drug side effects, a perceived lack of provider support, patient financial constraints, conflicts with the timing of treatment services, alcoholism and social stigma.

**Conclusions:**

Patient adherence to treatment is multi-factorial and involves individual patient factors, provider factors, and community factors. Addressing issue of LTFU during MDRTB treatment requires enhanced efforts towards resolving medical problems like adverse drug effects, developing short duration treatment regimens, reducing pill burden, motivational counselling, flexible timings for DOT services, social, family support for patients & improving awareness about disease.

## Introduction

Multidrug-resistant Tuberculosis (MDR-TB) is a major public health concern in many countries because of its difficulty in treatment and high costs. Resistance to anti-TB drugs can occur due to inappropriate treatment regimens, poor quality drugs, and inadequate intake of first line Anti-TB treatment [1]. Globally in 2013, an estimated 480, 000 people developed multidrug-resistant TB (MDR-TB) and almost 80% were from the European region, India and South Africa [2]. In India, there are about 64,000 MDR TB cases out of notified pulmonary TB cases [3]. Globally, overall treatment success for MDR-TB was 48% while 28% of cases were reported as being lost to follow-up (LTFU) or had no outcome information [2]. In India, for the treatment outcome reported in 2013 for cohort of 2010, treatment success rate was 48% with 18% LTFU [3]. Nagpur zone with one of the site (Sewagram) for drug resistant TB survey in Maharashtra was among the first sites in India to start MDR-TB treatment services and had 16 % treatment success rate, 11% death rate, 4% failure and 16% LTFU among 303 patients initiated on MDR-TB treatment till 2012 “S1 Fig”. LTFU is one of the key challenge in MDR-TB treatment; it also poses a public health threat because individuals who do not complete treatment are more likely to remain infectious and develop further resistance to existing drugs. World Health Organization (WHO) defined LTFU as “patient whose treatment was interrupted for two consecutive months or more” [4]. The findings of several studies conducted earlier identified high pill burden, long duration of treatment, unemployment, homelessness, history of the imprisonment, alcohol abuse, and baseline positive smear as independent predictors of lost to follow up [5,6,7]. As majority of these studies are conducted outside India, there is limited knowledge regarding factors for LTFU among MDR-TB treatment in India which has high burden of MDRTB cases. Therefore, more comprehensive understanding of patient and provider perceived barriers to treatment adherence is required to develop effective patient centered program strategies to reduce LTFU. A qualitative study was conducted to obtain better understanding of both patient and provider related factors for LTFU among MDR-TB treatment.

## Methods

### Study Setting

The study was conducted in seven districts of eastern Maharashtra with approximately 10 million populations. These districts are linked to Drug resistant TB centre (DRTBC) and intermediate reference laboratory at Nagpur. These patients were initially admitted at DRTBC for pre-treatment evaluation for approximately seven days and later received treatment at nearest MDR-TB DOT centre from their residence.

### Study design and sampling

Qualitative semi-structured personal interviews were conducted during August 2012–February 2013 with a purposeful sample of 20 MDR TB patients, who were reported LTFU among those registered for treatment during September 2007 to March 2012 and with 10 providers, which included government DOT providers, private treatment providers and community volunteers working as DOT providers for MDR-TB patients. Sampling strategy sought to ensure diversity of patients by age, sex, occupation, and residence in rural and urban areas and maximum variation in responses to the open-ended interview questions.

### Data collection and analysis

In-depth interviews were conducted in regional language using interview guide by the principal investigator along with the DRTBC Medical officer and District TB officer. Consent was obtained and interviews were electronically recorded. The interview guides for patients and providers were pilot tested and questions were readapted during concurrent analysis in accordance with a grounded theory methodological approach. Social cognitive theory was considered as a guiding framework for this study as it helps in understanding human actions, intuitions, motivations, and process that of behaviour change that determine adherence. The trained investigators who were trained in MDRTB national guidelines conducted minimum 45 min in-depth interviews. All study participants were interviewed at place convenient to them, which included patient residence, DOT provider’s clinics and government health centres. After each interview case-based memos were written which allowed us to capture different perspectives and in some cases participants were revisited to gather more insight and make comparisons between providers and patients reflections which enriched the data analysis. During the interviews we changed our questions and sequence in some cases to dig dipper and understand the relationships in patients and providers perspectives. Audio-recorded data from interviews of patients and providers was transcribed verbatim and transcribed into English. Codes and themes were developed concurrently with data collection. Direct quotes that illustrated important themes were extracted and presented in this manuscript.

### Ethical Considerations

The study protocol was approved by the ethics committee of the International Union for Tuberculosis and Lung Disease, (Paris, France) and India’s National Tuberculosis Institute (Bangalore, India) for ethical clearance. The CDC institutional review board determined CDC investigators were not engaged in human subject’s research as defined by U.S. regulations. Patients and provider participation in interviews were strictly voluntary and received no compensation. Individual verbal consent in Marathi and written informed consent from the patients and providers were obtained prior to interviews.

## Results

The study participants comprised of 20 MDR-TB reported as LTFU patients, between ages of 23 to 53 years of age and included 15 male patients and 5 female patients.12 patients were from urban area and 8 patients belonged to rural area. 12 participants were married of which three participants although married were not staying with their spouse and eight were unmarried. Among the 10 DOT providers recruited for the study 4 were female DOT providers and six were Male DOT providers. The age of DOT providers was between 25 years to 48 years and five DOT providers were urban area where as five were from rural area. Among the DOT providers, two were private practitioners, 2 community volunteers; four were government TB health visitors & two pharmacists from government sector.

The study participant’s in-depth interviews identified several factors that had impact on MDR-TB treatment adherence. Themes that emerged during interviews are summarised in Table 1 and verbatim quotes from participants are described below:

**Table 1:** Common themes that emerged during in-depth interviews with LTFU MDR-TB patients and treatment providers.

	Themes
**I. Medications related**	a)Adverse drug and treatment effects
	b)Long duration regimen
	c)Pain associated with daily injections
	d)High pill burden
**II. Service provider related**	a)Conflicting timings of job and treatment centres
	b)Behaviour of service provider
	c)Poor counselling
	d)Treatment facility access related
**III. Socio-economic factors related**	a)Stigma and discrimination
	b)Lack of family and social support
	c)Unemployment and financial constrains
**IV. Patient related**	a)Lack of awareness
	b)Myths and misbeliefs regarding disease
	c)Alcohol addiction
	d)Confidentiality issues

### Adverse drug and treatment effects

Six study participants reported adverse drug effects as an important barrier for treatment adherence. Both patients and providers reported side effects such as vomiting, severe headache, vertigo, restlessness, and psychiatric conditions. In these patients, adverse effects were an important reason to discontinue treatment as quoted by one of the patient [Patient 1, Male, 40years, Rural]:

“After taking medications I was neither able to sleep, nor was I able to move out. I had intense sweating… I felt like I would die today or tomorrow. Many don’t take these medicines because of this fear only.”

Depression, feelings of intense confusion, and suicidal thoughts were also commonly reported and linked to MDR-TB treatment medications as exemplified by a young male patient [Patient 13, Male, 26 years, Urban]:

“I was not able to see properly; I had itching all over my body. I had abdominal pain in the morning, and I could not sleep. I used to cry…I used to get up at midnight, talked like anything [patient was incoherent], not able to understand what is happening to me. My memory was going down. Sometimes I could not bear the pain, sometimes I had thought of suicide.”

The side effects experienced were also exacerbated by the quantity of pills, injections and lengthy time of MDR TB treatment. The daily regimen of 10–13 pills with injections for two years or longer made it difficult for two interviewed patients to complete treatment.

“On the first day after taking medications I felt like I was going up and down [experiencing nausea and dizziness]; I could not sleep the whole night. Everything was rotating, I felt as if I did not exist. Taking 12–13 pills was impossible for me…I am already weak, even when you utter the name of taking medicine, my head starts cracking.”

[Patient 10, male, 28 years, Rural]

### Support at the DRTBC and treatment Centres

Among interviewed patients, there were divergent opinions on the support and services provided by the DRTBC and local treatment centres. Two LTFU patients expressed that a lack of proper provider care was a relevant factor. Patient 9, perceived a lack of caring for his well-being by treatment staff:

“One staff sister [nurse] used to give injections. If I was late, she used to shout at me. Already I am lean and thin, the injection site used to bleed sometimes. Once my whole shirt was stained with blood.”

[Patient 9, male, 44 years, Urban]

Two patients also felt that they received inadequate information on the management of side effects or problems- this was also perceived as a lack of compassion and indifference from staff at treatment sites. One such patient, mentioned:

“When I told the staff there about vomiting, abdominal pain and itching over palms and legs after taking medications, they didn’t bother about it. They used to say ‘go to the medical college and tell them; there will be pain, still you will have to take medicines that we give.”

[Patient 11, male, 26 years, Urban]

The perceived lack of caring at government treatment facilities prompted three patients to seek treatment through private providers instead. Private practitioners were viewed as being more responsive to their needs than public providers are.

A private practitioner, DOT provider, stressed:

“Patients suffer from a lot of side effects like vomiting, headaches, skin rashes, but if his complaints are not addressed, he feels the drugs are not suitable for him so they visit private [providers]”

[Provider 10, Private Practitioner, Male,36 years, Urban]

### Conflict with the operating hours of treatment centres

Two patients had expressed their concern on the timing of the services in the treatment centres, particularly in urban regions and when it affected their employment.

Patient 19 noted that his centre’s “Timing was not proper. As a cleaner, I have to leave home at 6am in morning and work until 2pm in afternoon; their centre [the government DOT centre] is only open until 12 noon. My duty timing and treatment centre timing were not matching so I left treatment. Now I take treatment from a private provider. They give treatment month-to-month [monthly instead of daily] so I am able to do work and be happy.”

[Patient 19, Male, 38years, Urban]

Government providers also recognized the timing constraints of centres. Although they desired to make their hours more accommodating to patients, there was difficulty in doing so because of their numerous other responsibilities. Facilities with few personnel in particular faced this challenge. Provider 6 who works in a small rural health centre expressed her frustrations:

“I have to work for TB, Leprosy, and immunisation programs- everything. How much time can I give? Some patients were coming in the evening, I scolded them- I have to prepare food, I too have to look after my family. How can I give time during the evening or night?”

[Provider 6, Government DOT provider, Female, 46 years, Rural]

### Alcohol abuse

Although most interviewed patients themselves did not note alcohol addiction as a factor for not adhering to treatment, alcohol abuse was expressed as a major barrier to treatment adherence noted by their providers. Alcohol abuse resulted in not only missed MDR TB treatment doses and other scheduled appointments, but in patients also being unreceptive to counselling and treatment adherence messages by providers. One provider described these typically male patients as being an “army of one” because of their social isolation and difficulties in being receptive to counselling. Provider 9 noted his experiences with such a patient:

“Most of them are addicted to alcohol consumption. In addition, most are adamant, they do not listen. One such patient threw chapels (shoes) at me while he was under the influence of alcohol.”

[Provider 9, Government DOT provider, Male, 30 years, Urban]

Because alcohol abuse was intertwined with treatment non-adherence, providers suggested that relationships be developed with alcohol control programs.

### Social Stigma and Discrimination

Themes of social stigma and discrimination as barriers to completing MDR-TB treatment also emerged during interviews with patients and providers. Three patients reported being socially isolated and discriminated against for being infected with MDR-TB. This fear of discrimination directly interfered with MDR-TB treatment and activities to promote adherence. Patients did not want health workers to visit their home for adherence counselling and did not want to attend their local treatment centre due to a potential disclosure of their disease. In particular, addressing issues of social stigma for unmarried women infected with MDR TB was challenging for providers. A private DOT provider noted that she needed to prioritize the counselling of the parents:

“They [the parents] were worried of her marriage [prospects] and they didn’t want to disclose the disease also. They wanted to hide this; they did not want anyone to know about her disease… You have to counsel the relatives as well. It is almost as important [as counselling the patient]. I counselled her parents at that time even when they used to say ‘We can’t see her in this way, and we have to stop the treatment.’

[Provider 5, Private practitioner, Female, 45 years, Urban]

Fear of discrimination not only led to patients and family members not wanting to disclose about their treatment, but also in some patients did not want health workers to visit their home or did not want to take treatment at a nearby centre due to a fear of potential disclosure about their disease and MDR-TB treatment in their catchments areas. Patient 15 living in slum mentioned:

“They talk about me differently and gossip about my disease, I have a family to look after. My daughter is yet to be married.”

[Patient 15, male, 47 years, urban slum]

### Family and Social support

For other younger married women who lacked supportive spouses or in-laws, support came from their maternal mothers. Support typically was in the form of verbal encouragement to take medicines regularly, the provision of food, and encouragement to focus on ones health despite the difficult and lengthy treatment.

Patients who lacked strong networks of family and social support had were more prone for LTFU. Two married female patients lacked support from her husband, who left her alone to be cared for by her mother (maternal). Patient 17 noted that:

“My husband spend money on drinking alcohol, but didn’t give a single rupee for my medical expenses…he never cared...I used to work during illness also…my health was deteriorating. I called my mother one day and she hospitalised me. I would not have survived living with him.”

[Patient 17, female, 37 years, Urban]

### Myths and Misbeliefs

Lack of proper awareness of disease and its treatment also influenced treatment. Myths and misbeliefs among patients who did not complete treatment were identified. A male patient from rural area mentioned:

“I don’t have TB, This is something else. This is external spirit influencing me. I went to goddess [local faith healer].she gave me ash. Since 2–3 months I am taking that daily...I feel ok now”

[Patient 6, Male, 35years, Rural]

A perception of MDR-TB treatment being more harmful to one’s health was particularly evident in a patient who was LTFU. Patient 1 specifically noted:

“I mean I had no relief [from MDR TB treatment]. Even after taking pills and many injections I had no relief- what is the use then? It is better to die at home rather than to take these medications.”

[Patient 1, Male, 40years, Rural]

## Discussion

**Fig 1:**
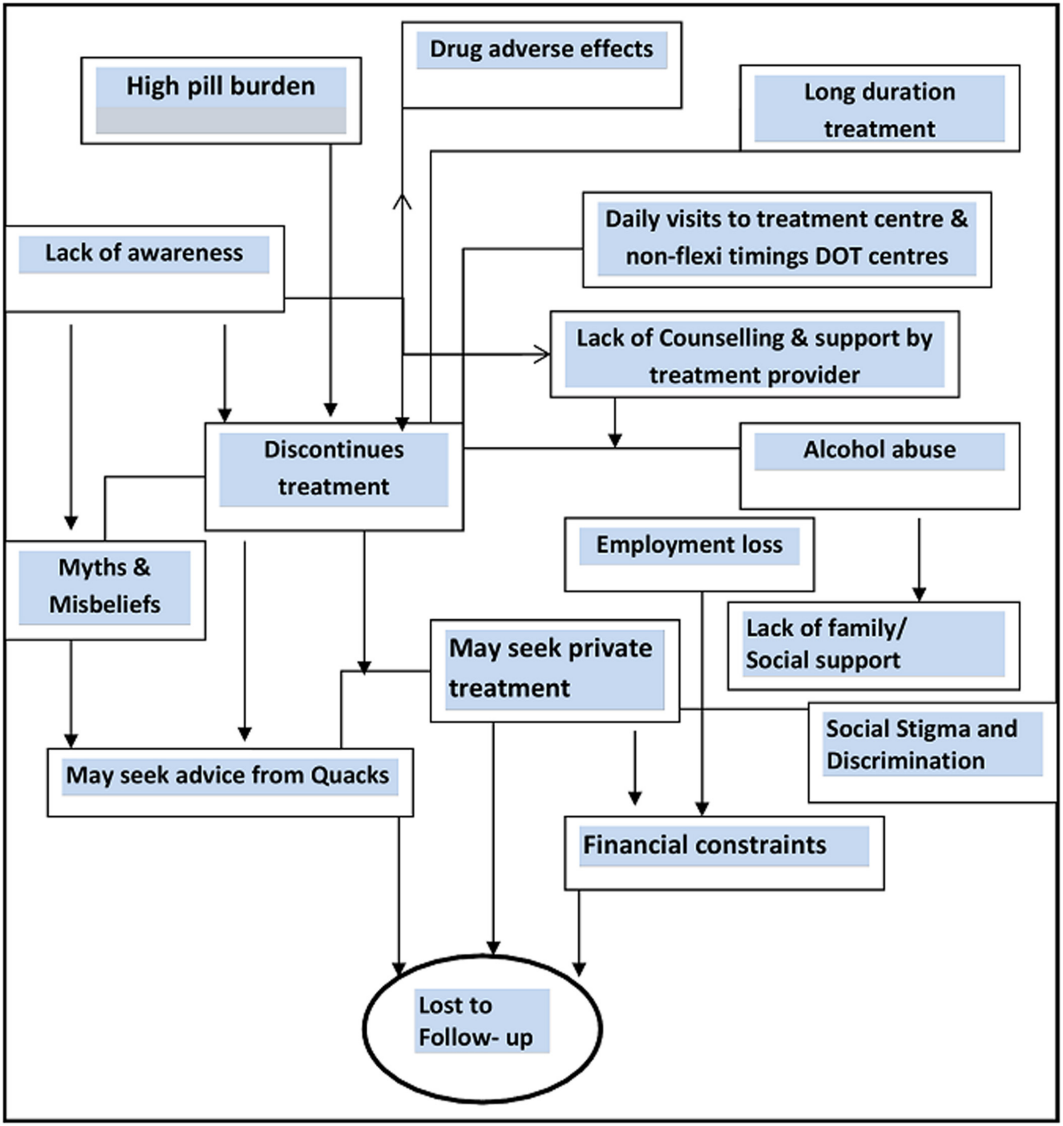
Web of multifactorial causation for lost to follow- up among MDR-TB Treatment, Nagpur.

This study focused on qualitative interviews with both patients and treatment providers. Similar to a previous quantitative study on TB adherence in a neighbouring district [8], our findings reveal that factors related to LTFU among MDR TB treatment are multi-faceted as shown in “Fig 1”. These ranged from patient experiences with treatment, to support from providers and family members, and to the social circumstances, characteristics, and self-beliefs of patients. The promotion of MDR-TB treatment adherence, therefore, will require a multi-faceted targeted approach.

Our finding of a negative relationship between experiencing adverse effects to MDR-TB treatment and adherence is consistent with previous studies [9, 10, 11]. Vega P et al. described psychiatric issues in the management of patients with MDR-TB including depression and anxiety [12], two issues vividly described during patient interviews. A shorter duration of MDR TB treatment (currently 24 months) [13] and addressing psychiatric issues in patients that arise during treatment [12] have been recommended as potential strategies to improve adherence.

Alcohol abuse by patients presents another substantial barrier in MDR TB adherence and has been noted elsewhere by studies of resistant TB treatment adherence [7, 14, 15, 16]. Alcoholism interferes in the regularity of treatment appointments and limits the effectiveness of the provider support and the counselling process. In this study, interviewed providers have suggested developing linkages with alcohol addiction program. This strategy has also been promoted previously and requires additional consideration [16].

Our finding of a perceived lack of support from government providers and the inconvenient operating hours of facilities were reported previously as barriers to treatment adherence in a study in an urban setting of Delhi, India [17]. Although flexible centre hours have been recommended by the local TB control program, having sufficient providers trained to administer injections for MDR TB treatment as well as having limited personnel in smaller facilities constrain this option.

In our study, some patients at particular risk for not completing treatment were those with direct conflicts between their job responsibilities and treatment centre hours. These were patients who were their household primary earners and who worked on daily wages. The inability to work due to treatment side effects, the relationship between financial constraints and having adequate nutrition, and the threat of job loss were all intertwined factors that present challenges for patients to complete treatment. Targeted approach of identifying these vulnerable individuals and developing programmatic strategies to provide comprehensive support is necessary to improve treatment adherence.

## Limitations

Considering the differences in programmatic implementation in different regions, the socioeconomic variations, and our findings may not be necessarily generalised to other sites, moreover reasons for lost to follow-up may differ in different settings. Secondly as the study involved in-depth interviews of patients whose outcome were reported and providers, recall bias more distal from time of data collection could exist particularly for patients and treatment providers. To minimize the recall bias participant responses were also triangulated with treatment providers. Interviewers were also trained to probe during interviews to facilitate an accurate recall of events.

## Conclusion

Adherence to MDR-TB treatment and LTFU among MDR-TB patients remains a pressing public health problem to address. Addressing issue of LTFU during MDR-TB treatment requires enhanced efforts towards resolving medical problems like adverse drug effects, developing short duration treatment regimens, reducing pill burden, motivational counselling, flexible timings for DOT services, social support, family support for patients & improving awareness about disease. Further implementing research is needed for devising strategies to address these issues and to document practices for improvement in adherence to MDR-TB treatment.

## Supporting Information

S1 Fig Treatment of MDRTB cases Nagpur. (TIF)
