# Supplementary figures and images for: Patient and Provider Reported Reasons for Lost to Follow Up in MDRTB Treatment: A Qualitative Study from a Drug Resistant TB Centre in India
Source: PLoS One. 2015 Aug 24;10(8):e0135802. doi: 10.1371/journal.pone.0135802 (PMC4547708; doi:10.1371/journal.pone.0135802)

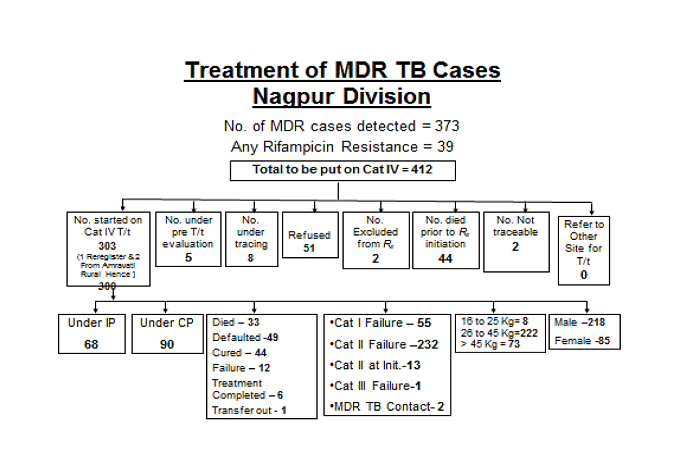

Supplement: S1 Fig — (TIF) [file pone.0135802.s001.tif]
